# Trust and Biomedical Research Engagement of Minority and Under-Represented Communities in Mississippi, USA

**DOI:** 10.3390/ijerph20021592

**Published:** 2023-01-16

**Authors:** Donna Antoine-LaVigne, Traci Hayes, Marty Fortenberry, Evidence Ohikhuai, Clifton Addison, Sam Mozee, Dorothy McGill, Mangle L. Shanks, Christopher Roby, Brenda W. Campbell Jenkins, Paul B. Tchounwou

**Affiliations:** 1 RCMI Center for Health Disparities Research, Jackson State University, Jackson, MS 39217, USA; 2 School of Health Professions, University of Southern Mississippi, Hattiesburg, MS 39406, USA; 3 Jackson Heart Study Graduate Training and Education Center, School of Public Health, Jackson State University, Jackson, MS 39213, USA; 4 Mississippi Urban Research Center, Jackson State University, Jackson, MS 39201, USA; 5 Innovative Behavioral Services, Ridgeland, MS 39157, USA; 6 Shanks Health Housing, Employment, Education & Training Services, Inc., Jackson, MS 39289, USA; 7 Mississippi Health Alliance, Byram, MS 39272, USA

**Keywords:** trust, community engagement, African American, research participation, RCMI programs

## Abstract

Trust is critical to the development and maintenance of effective research collaborations and community engagement. The purpose of this study was to assess the current attitudes and level of trust pertaining to health research among residents of Central Mississippi, the priority health region for the Research Centers in Minority Institutions (RCMI) Center for Health Disparities Research (RCHDR) at Jackson State University. The cross-sectional study was conducted from November 2021 to April 2022. The data were analyzed using descriptive statistics carried out by SPSS statistical software. A total of 146 participants responded to the survey. The participants were predominately African American (99%) and female (75%). Historical research studies, the researchers’ qualities, and potential benefits from participation were factors affecting the level of trust in the research process. Ninety percent (*n* = 131) expressed that it was important to be involved in the research process, and 98.5% (*n* = 144) agreed that discussing the research findings with the participants was important for establishing trust in the research process. While trust in the research process does not guarantee participation, trust is a precursor for those who decide to engage in health disparities research. Key findings will be integrated into the RCHDR research agenda to foster further development and implementation of innovative community-based participatory research toward the control and/or prevention of diseases that disproportionately affect minority and under-represented populations in Mississippi.

## 1. Introduction

Trust, according to Webster’s dictionary, is defined as confidence, reliance, or resting of the mind on the integrity, veracity, justice, friendship, or other sound principles of another person. Trust is also defined as one’s belief that an entity (person, group, or organization) will act in one’s best interest. Trust is critical to the development and maintenance of effective collaborations and community engagement. It improves satisfaction with the research study, as well as results in better study outcomes [1,2,3]. Research mistrust is defined as the belief of the study participant that his/her needs are insignificant to those of the study or the researcher as evidenced by conditions such as withholding valuable information, risks outweighing the benefits, or using data to damage individuals or communities [3,4,5,6]. Over the years, researchers have documented the lack of trust in the African American community and how it is demonstrated through their limited participation in health and medical research [5,7,8,9]. It is often reported that this lack of trust is partially due to the “Tuskegee Syphilis Study” conducted from 1932 to 1972 by the Centers for Disease Control and Prevention and the United States Public Health Services [10,11,12]. Recognized as one of the most egregious studies in history, this unethical research has greatly limited African Americans’ willingness to participate in biomedical research. The treatment of the male participants and the lack of transparency during the Tuskegee Study resulted in mistrust in research that continues to deter African Americans from participating in research to this day [8,9]. It has been documented that the acclaimed national news correspondent Harry Reasoner described the “Tuskegee Study” as an experiment that “used human beings”. This was performed in a manner that was not in the best interest of the participants [10]. 

Consequently, the mistrust for medical research and scientists was rooted in the African American community prior to the Tuskegee Syphilis Study. The lack of trust in health and medical research among African Americans can be traced to the incidents of medical experimentation and mistreatment during the pre- and post-civil war slavery period [13,14]. Before the syphilis study, there was research abuse that involved Dr. J. Marion Sims who was known as the father of modern gynecology. From 1835 to 1846, Dr. Sims used three enslaved African American women as subjects in his research to develop a procedure to repair vescio-vaginal fistulas. The women underwent up to 30 painful operations without the use of anesthesia. Only after he perfected his technique on enslaved women did he attempt the procedure on white women with painkillers [15]. Such studies have led to decades of continued mistrust of research by African Americans [10]. 

It is widely accepted that health research that includes members of the priority population as active contributors to the research process results in improved study outcomes and adherence to the findings [2,5,6,8,9,10,16,17,18,19]. Efforts to understand how diverse populations engage in research are significant to the delivery of patient-centered care [18]. It is predicted that the African American population will increase at a rate of 40% over the next 40 years; because of this, the attitudes of this group toward research are important for the development of therapies and treatments to address their growing health concerns [9]. Despite national efforts by the National Institutes of Health to increase the participation of diverse and minority groups in health and medical research, African Americans’ participation continues to be a challenge [5,10,20]. Numerous studies highlight the lack of participation in research trials among African Americans and the limitations it presents when attempting to generalize findings toward the greater population [6,14,21,22,23,24]. African Americans express other concerns causing their hesitance about participating in research studies, including researcher bias, the possibility of being unfairly treated, being exposed to unfavorable conditions, and not experiencing any improvements in their outcomes [8,25,26,27]. Smirnoff and colleagues [5] associated several factors with the lack of minority participation in research but assigned mistrust as the most pertinent factor among the group. Various researchers have agreed that trust in the research and the research institutions is significant for participation and engagement in research [1,2,8,9,28]. 

In a foundational study on community trust, Corbie-Smith [10] acknowledged that participants were aware of the mistreatment that occurred during the Tuskegee Syphilis Study. The study participants revealed that their lack of trust in the medical community was a major impediment to research participation and shared experiences of exploitation to validate their views of distrust [10]. According to Glover, Sims, and Winters [28], the lack of trust typically results in dissatisfaction. The researchers discovered that trust was paramount for patient satisfaction and that it involved examining previous interactions with healthcare professionals. In their study to examine the differences in trust levels among Latinx and African Americans and their willingness to participate in nontherapeutic research, Thetford and colleagues [9] found that African Americans have the same openness to engage in research as their white counterparts. They reported that 75% of the 553 participants had positive views of health-related research. Nonetheless, the researchers acknowledged that low participation among African Americans may have been dependent upon their views of treatment due to their race and ethnicity along with having distrust in the medical procedures. 

The Jackson Heart Study (JHS), a twenty-year longitudinal study to examine cardiovascular disease among the largest cohort of African Americans in the United States, demonstrates how incorporating community engagement priorities can establish trust and maintain the interest of the community in research. The participants’ trust strengthened through community engagement has resulted in high participant recruitment, participant retention greater than 80%, and the improvement in specific health outcomes among African Americans in the study [8,13,28]. Among the lessons learned by the principal investigator and JHS Community Outreach Center was that the mistrust in the healthcare system continues to be a barrier for African Americans participating in biomedical research studies. A major recommendation to overcome such barriers was to build trust in the initial stages of the study among potential participants, their families, and the communities in which they live. This included developing and implementing specific activities of interest to the population. The JHS cohort and community expressed interest in designing outreach literature, reviewing, and modifying consent forms using easily understood lay language, and suggesting other forms of engagement. Despite implementing these efforts, the process of building and maintaining trust remained difficult [13]. Other researchers suggest including trust-building methods in study recruitment and implementation [9,28]. These include explaining study procedures in a clear and plain format for greater understanding, engaging race/ethnicity-matched study coordinators along with acknowledging past mistreatment of minority communities while assuring them of the safeguards for their well-being. A mixed methods study to map the experiences of trust revealed the need for immediate actions to improve trust within research partnerships [29]. There is hesitancy among researchers to recruit African Americans due to their mistrust in the process; believing that such reluctance may slow down the completion of consent forms or result in the community members dropping out of the study before its completion [8]. Consequently, African American communities may unknowingly miss beneficial study findings and therapies tailored for the population. The research on participants’ attitudes and perceptions of research is increasing in importance [5,6]. Therefore, community members’ attitudes towards and the level of trust in the research process remains a critical area for investigation, which can lead to actionable strategies that increase participation in the health disparities research. Health researchers have a responsibility to better understand factors such as mistrust that contribute to the low participation rates among minorities in health research [7]. Historically, the state of Mississippi has been burdened with some of the nation’s worst health outcomes [30]. Its residents suffer disproportionately from chronic illnesses, limited healthcare access, and the woes associated with the social determinants of health, particularly the African American population [31]. In the Central Mississippi counties of Hinds, Rankin, and Madison, African Americans make up 73%, 38%, and 21%, respectively, of the population and suffer from the highest rates of diseases and illnesses among the residents [31,32]. For these reasons, medical studies such as the JHS are seen as necessary for the community’s well-being. Thus, securing the participation of those at high risk of many of these conditions remains essential for advancements. 

The research on the importance of participants’ attitudes and perceptions of research is increasing. The current study is novel because it relies on the principles of community-based participatory research to assess the influencing factors for trust and participation in research. Acquiring a greater understanding of the intricacies involved in research trust as a means of improving African Americans’ engagement in research is the overarching goal of this study. Its purpose was to assess the current attitudes and level of trust pertaining to health research among residents in the Central Mississippi tri-county areas of Hinds, Rankin, and Madison, the priority health region for the RCMI Center for Health Disparities Research (RCHDR) at Jackson State University. 

## 2. Materials and Methods

Applying the specific principles of the CBPR model, the current study sought to understand the attitudes towards and levels of trust for health disparities research among individuals residing in three Mississippi counties served by the Research Centers in Minority Institutions (RCMI) Center for Health Disparities Research (RCHDR). The RCHDR relies on the support and engagement of its community members and seeks to share research findings and relevant health education materials with the lay community. Therefore, to establish a community-supported research agenda, the RCHDR Community Engagement Core (CEC) interacted with community members to better understand how the residents’ felt about the health disparities research being conducted in their communities. The following principles of the CBPR model were utilized throughout the study. Principle one, the community was informed of the study and recognized as a unit that should have input. Their input helped to address principle 5, ensuring information garnered and shared was relevant and there was value added for the community. Finally, principles 7 and 9 were addressed by keeping the community involved in the evolution of the study through an iterative process allowing for feedback on instrument development and validation and dissemination of findings.

### 2.1. Participants

Adult men and women 18 years of age and older who lived in Mississippi and resided in any one of the target counties of Hinds, Madison, and Rankin, which are served by the RCHDR, were eligible to participate. 

### 2.2. Study Design and Process

The RCHDR CEC conducted a cross-sectional study from November 2021 to April 2022. The study was approved by the University’s Institutional Review Board (#0014-22). The purposive sample of 150 individuals residing in the tri-county area was established a priori. A snowball strategy was utilized to finalize the study sample where each participant was asked to identify other potential survey takers or to share the link with those residing in the selected counties. Survey participants were recruited by the researchers, as well as members of the RCHDR Community Advisory Board via word-of-mouth and text messaging. Other recruitment activities included: (1) Posting the study information on the RCHDR website; (2) Emailing the information to members listed on the University and partnering community organizations’ listservs; (3) Placing printed surveys with consent forms on-site at partnering organizations and agencies. 

The survey and consent forms were administered via online and printed formats. The consent forms included the following statement, “This study is completely voluntary, and you have the right to choose to or not to participate. If you choose to participate, you can stop engagement at any time or refuse to answer any one of the survey questions without fear of penalty”. The consent form was displayed online or as a hard copy. The consent form was signed before starting the survey. 

The initial approach to the distribution of the trust survey required posting the survey link on the Center’s website. After two months, there were only 40 completed responses. The CEC research team consulted with its Community Advisory Board (CAB) to identify strategies to increase survey completion. While different efforts were employed, such as emailing individuals, introducing a text message that included the survey link, which was sent to phone contacts, helped the research team meet the participation goal. 

### 2.3. Survey Instrument

The community trust instrument was adapted and customized from a previous instrument utilized by the JHS Community Engagement and Outreach Core. Three authors drafted the structured questionnaire, which was revised after feedback from another expert from the JHS at Jackson State University. The survey was created in Qualtrics XM. The survey was piloted with the Community Advisory Board (*n* = 15) to ensure validity and interpretability. The pilot provided feedback on the readability and ease of answering the questions and the length of time needed to complete the survey. The recommendations included updating the questionnaire to include instructions on answering a “ranking of answers” question when responding through the online form and ensuring printed copies of the survey would be available for requesting partner organizations. Once the survey was updated, another review was conducted, and the final survey was approved. The final version of the 30-item questionnaire included questions about the demographics of the participants and their attitudes and views toward health research. Questions 1–6 collected demographic data that included age, gender, race and ethnicity, and place of residence. Questions 7–9 asked the participants’ level of participation in community activities, including research activities, and the type of participation. These were multiple-choice and open-ended questions. Questions 10–29 relied on a 5-point Likert scale, with answers ranging from ‘extremely important to ‘not important at all’, ‘extremely likely to ‘not at all likely, or ‘extremely trusted’ to ‘no trust at all’. Questions 10–16 solicited the participants’ level of trust in the research personnel and how certain factors influenced their trust in the research team. Questions 17–25 included questions about the participants’ views of the community’s participation in the research process. Questions 26–29 asked about their trust in the COVID research and their willingness to participate in the COVID research. The last, question 30, asked the participants to rank the importance of health issues to be considered for future research endeavors. The choices were determined by the chronic illnesses listed on the Mississippi Department of Health’s website. Refer to Appendix A to review the RCHDR CEC community trust survey instrument.

### 2.4. Data Analysis

This study used a descriptive research design. The data were analyzed using descriptive statistics carried out by SPSS statistical software (https://www.ibm.com/spss). Categorical variables were described using frequencies and percentages. Descriptive statistics are specific methods that enable the researcher to use, calculate, describe, and summarize collected research data in a logical, meaningful, and efficient way. Descriptive statistics enable the researcher to present data numerically in the document and/or in its tables, or graphically in figures and charts. Descriptive statistics were employed to present the characteristics of the population under investigation and allowed the researcher to communicate the results of the analyses to the local community and other interested parties and stakeholders. Descriptive statistics are appropriate for presenting the data to the lay community [33]. Data were presented as frequencies and percentages, revealing the number of occurrences, and providing some context for comparisons of groups, as well as an understanding of how groups within the target population perform [33]. 

## 3. Results

A total of 146 participants responded to the survey: 22% self-identified as men, 75% as women, and one individual selected non-binary or third gender. Approximately 68% were between the ages of 35–64 years of age. The survey participants were predominately African American (99%), and two of the participants were white. Demographic details are presented in Table 1.

Forty percent had no previous experience in research or community engagement. The remaining 60% had one or more years of involvement in research or community activities. The largest group of participants (43%) had planned community activities and 35% were first-time participants. Table 2 displays the type of research and community engagement.

More than 50% (*n* = 108) of survey respondents expressed that the historical Tuskegee Study was important when considering participating in research. Nearly 94% (*n* = 136) of the survey participants agreed that a researcher’s prejudice can affect their trust in the research and 96% (*n* = 141) acknowledged that the researcher’s attitude was important for gaining their trust. Most of the participants, 98.50% (*n* = 144), agreed that the researcher’s knowledge was important in establishing trust in the research process. Nearly 80% (*n* = 116) of the respondents would like the researchers to look like them. Table 3 displays the level of trust among participants. 

Ninety percent (*n* = 131) expressed that it was important to be involved in the research process, and 98.50% (*n* = 144) agreed that discussing the research findings with the participants was important for establishing trust in the research process. Additionally, having the support of the community leaders, including pastors and health advocates, would increase the level of trust among 92% (*n* = 131) of the respondents. Ninety-six percent (96%) believed that the research findings were likely to benefit their communities. Table 4 displays the level of importance the participants assigned to the benefits associated with community research. 

## 4. Discussion

The purpose of this study was to assess the current attitudes and level of trust about health research among residents in the Central Mississippi tri-county areas of Hinds, Rankin, and Madison, identified as the focus area of biomedical, socio-behavioral and/or clinical research conducted by the Research Centers in Minority Institutions (RCMI) Center for Health Disparities Research at Jackson State University. The study sought to identify factors that could influence community trust in the research process and research engagement. While trust in the research process does not guarantee participation, trust is a precursor for those who decide to engage in the health sciences research process. 

The results of this study serve to emphasize the importance of trust for study participation, particularly among African American communities. The responses of the participants signified that establishing trust involved several factors. They are the following:

1. Researcher’s attitude;
2. Researcher’s race;
3. Researcher’s knowledge;
4. Participant engagement;
5. Sharing study findings;
6. Benefits to the community;
7. Community leaders (stakeholder) engagement;
8. Incentives/resources garnered from participation.

Trust is instrumental in inspiring community members to participate in research activities. Many of the participants had no previous experience in research or community engagement, and more than half of the survey respondents expressed that the historical Tuskegee Study was important when considering whether they will participate in the research. Many of them are still mindful of the fact that community members participating as research subjects had their rights violated. In addition, a variety of other ethical issues, such as informed consent, racism, paternalism, unfair subject selection in research, maleficence, truth-telling, and justice, were synonymous with that study. While about two-thirds of them had one or more years of involvement in research or community activities, less than half of the participants had planned community activities experience, and one-third of them were first-time participants.

Most of the survey participants agreed that a researcher’s prejudice can affect their trust in the research and most of them acknowledged that the researcher’s attitude was important in gaining their trust. So, there are sometimes caution warning signs aroused when the rigor, objectivity, and validity of the researchers’ work come into question. In such cases, researchers’ work is believed to be driven more by their own vested interest or personal agenda.

The participants also indicated that they believed the researcher’s knowledge was important in establishing trust in the research process. In biomedical research, in particular, the absence of trust is a serious threat to the success of any research enterprise. The majority confirmed that they would like the researchers to look like them.

Most of the participants understood that it was important to be involved in the research process, and they believed that discussing the research findings with the participants was important for establishing trust in the research process. Scholars believe that trust in communities can also translate to other types of successes and accomplishments, such as stronger volunteerism, healthier residents, as well as economic prosperity. The participants in this study believed that having the support of the community leaders, including pastors and health advocates, would increase the level of trust. Participants were convinced that the research findings were likely to benefit their communities.

### 4.1. Historical Research

The research suggests that African American participation in health research and clinical trials has been limited [4,6,12]. Despite this conclusion, the engagement of African Americans in foundational research studies, at times through involuntary participation or under unethical pretenses, has contributed significantly to medical knowledge [24]. The Tuskegee Syphilis Study or the gynecological research studies conducted by Dr. Sims are often viewed as justification for African Americans’ angst and apprehension for medical and health research. In the current study, more than 50% of the participants expressed that the historical Tuskegee Syphilis study was important when considering participation in research and along with other considerations, such as the researchers’ qualities, research implementation, and research gains, influenced their level of trust in the research process.

### 4.2. Researchers’ Characteristics

Researchers, such as Thetford and colleagues [9] and Peek and colleagues [25], found that racial bias and cultural insincerity were viewed negatively for patient trust and engagement. The first was the race and ethnicity of the researcher. Approximately 80% of the predominately African American participants wanted to see researchers who look like them and felt it was important for increasing trust in the research. Second, the participants identified that the researchers’ attitudes toward them influenced their level of trust in the research process. Nearly 74% (136) of the participants believed that the researchers’ own biases and prejudices affect the study and its outcomes. The third consideration for ensuring trust was the researchers’ knowledge. This was framed as the researchers’ understanding of the condition, disease, or illness, as well as how the health issue was affecting the participating community. According to the work of Peek and colleagues [25], medical competence and skills were recognized as a primary source of trust for the participants. When expected health outcomes were met, their patients’ confidence and trust in the physician and the process increased [9,25].

### 4.3. Research Implementation

Improving the level of trust in the research process with the hopes of increasing participation requires transparency and a willingness to disclose truthful details regarding risks and benefits [27]. This was also suggested by Bonner et al. [4] and Peek et al. [25]. The current study reveals that approximately 90% of the participants agreed that it was important to allow the study volunteers to engage at various stages of the research process, such as idea generation through to the dissemination of the findings. Ninety-five percent of participants acknowledged that sharing the study findings with the study participants was important for improving trust. It was revealed that 92% of the participants believed having the support of the community leaders, such as pastors or health advocates, was important for the trustworthiness of the research and would improve community engagement in the process. According to Bronner and colleagues [4], engagement in research was more likely when the study information was presented by the pastor or clergy leadership and when it was not, the participants were less likely to trust the project.

### 4.4. Research Gains

Nearly 70% of participants agreed that financial incentives were important, and 57% agreed that some form of compensation other than money would increase their participation in the research; noting that this gesture demonstrates that the researchers value and respect the participants’ time and commitment. As the number of projects implemented in the community increases, the public is becoming more aware of the financial investments and gains for the researchers [26,27]. In the present study, 95% of participants felt it was important to provide sustainable benefits to community members and especially those who were involved in the research.

Overall, the survey participants expressed trust in the research process and their attitudes suggested future engagement in trustworthy research projects led by credible researchers.

### 4.5. Limitations

The current study provides results for a very small sample of the predominately African American population in select counties, and therefore, may not be generalizable to other racial groups or those who do not reside in the study area. Recruitment bias may affect the generalizability of the findings. Utilizing the snowball strategy for recruitment can attract participants from friend groups with similar attitudes and views pertaining to a topic. As a cross-sectional study relying on an online survey, willing participants without internet access were unlikely to complete the questionnaire and we were unable to expound on various points of interest, including personal reasons for the trust in the research process. For this study, not all participants responded to every survey question, so there are missing data.

## 5. Conclusions

The state of Mississippi has historically and continuously had the worst health indicators in the nation and its African American residents, who are disproportionately affected by many diseases [30,31] could benefit greatly from the outcomes of health research. Securing the research participation of individuals at high risk of these illnesses is essential to improve their health outcomes. Several factors are important to increasing trust in the research process. The findings of this study aid in our understanding of some factors that are important for establishing trust in research among specific African American populations in the state of Mississippi. This information can help to foster more trustworthy and lasting researcher and community relations. It highlights the great interest among this group to engage in efforts to help their communities, and that research is viewed as one of those efforts. It demonstrates that this population has a positive outlook on research and has the willingness to participate if it is inclusive, sharing in the decision-making and implementation process, and is beneficial, adding value and assets to the community.

The study supports policies that will inform and train minority communities to work on the research studies and establish standards for community incentives and sustained resources. Policies and programs should focus on ensuring the next and future generations of minority researchers in addition to providing tailored learning opportunities that prompt and sustain their involvement. This would include ensuring versions of the protocol and findings are accessible and translated for their comprehension. Research sponsors may find it beneficial to ensure trusted community-based organizations and representatives are primaries on the projects and recipients of funding, guaranteeing that resources are assigned to facilitate the execution of their roles and tasks. The study findings support the importance of the relationship established between the researchers and the community members. The researchers are the face of the process and the first point of interaction to foster the trust required to engage the community members in research discovery. There is a growing consensus that researchers who represent the racial and ethnic distinctions of the study participants can improve trust and create a sense of comfort among those participants [7,24]. Researchers have agreed that it is necessary to garner the trust of the priority population to improve community engagement in the research process [1,2,8,9,28]. The RCHDR will integrate these key findings to ensure that there is a bidirectional relationship between its investigators and the community and that the community members are involved in shared decision-making regarding the development and implementation of innovative research aimed at improving minority health and reducing health disparities in the three-county area of Central Mississippi. Additionally, RCHDR will strengthen the training of its investigators in community-based participatory research principles, transparency, and cultural competency.

## Figures and Tables

**Table 1:** Demographic characteristics of the participants.

Participants	Frequency (*n*)	Percentage
Men	32	22.07%
Women	112	77.4%
Non-binary/Third gender	1	0.69%
18–20	1	0.68%
21–34	23	15.75%
35–44	33	22.60%
45–54	30	20.55%
55–64	36	24.66%
65–74	22	15.07%
75–84	1	0.68%
Black	144	98.63%
White	2	1.37%
Hinds	100	68.49%
Madison	16	10.96%
Rankin	11	7.57%
Not specified	19	13.01%

**Table 2:** Type of research and community engagement activities.

Participants	Frequency (*n*)	Percentage %
Years of Participation in research and/or community engagement		
0 years	58	40.0%
1 year	11	7.59%
2–4 years	24	16.55%
5–8 years	19	13.10%
9–11 years	5	3.45%
12 years or more	28	19.31%
Are currently serving as		
RCMI Community Advisory	16	18.39%
Community Health Network (CHAN)	10	11.49%
Community member/local resident/stakeholder	61	70.11%
Role(s) in research and/or Community engagement		
Planning community outreach activities	43	22.28%
Outreach for recruitment	27	13.99%
Hosting community health fairs	29	15.03%
First-time participants in research studies and/or activities	35	18.13%
Design/present health education messages to address health disparities in the African American community	26	13.47%

**Table 3:** Level of trust among the study participants.

Questions	Extremely Important		Very Important		Somewhat Important		Not So Important		Not at All Important	
How Important	*n*	%	*n*	%	*n*	%	*n*	%	*n*	%
Q10. Is the Tuskegee Syphilis Study to your decision to participate in research?	45	31.47%	36	25.17%	27	18.88%	17	11.89%	18	12.59%
Q11. Is the researcher’s attitude in gaining your trust?	65	44.52%	60	41.10%	16	10.96%	3	2.05%	2	1.37%
Q12. Is the race of the researcher to your decision to participate?	29	19.86%	41	28.08%	43	29.45%	20	13.70%	13	8.90%
Q13. Is the researcher conducting the research to look like you?	31	21.23%	43	29.45%	42	28.77%	19	13.01%	11	7.5%
Q14. Is ethnicity and race to your trust of the researcher?	30	20.55%	44	30.14%	46	31.51%	17	11.64%	9	6.16%
Q15. Is the researcher’s knowledge to your trust in the research process?	87	59.59%	44	30.14%	13	8.9%	2	1.37%	0	0%
Q16. * Is the researcher’s prejudices (biases) affecting your trust in the research?	53	36.55%	54	37.24%	29	20.0%	7	4.83%	2	1.38%
Q17. Is it to be involved in the research process?	45	31.03%	54	37.24%	32	22.07%	14	9.66%	0	0%
Q18. Is discussing the research findings to your trust in the process?	61	42.36%	65	45.14%	14	9.72%	4	2.78%	0	0%

* For Q16, the question was, “How likely are the researcher’s prejudices (biases) to affect your trust in the research? Responses were “extremely likely” = 53 (36.55%); “very likely” = 54 (37.24%); “somewhat likely” = 29 (20%); “not so likely” = 7 (4.83%); “not likely at all” = 2 (1.38%).

**Table 4:** Benefits of community research.

Questions	Extremely Important		Very Important		Somewhat Important		Not So Important		Not at All Important	
How Important	*n*	%	*n*	%	*n*	%	*n*	%	*n*	%
Q21. Is it that the community benefits from the research?	73	52.14%	59	42.14%	7	5.0%	1	0.71%	0	0%
Q22. Is a community leader’s support of the research for your participation?	46	32.39%	62	43.66%	23	16.20%	9	6.34%	2	1.41%
Q23. Is a friend’s support of the research to your participation in research?	35	24.82%	50	35.46%	35	24.85%	17	12.06%	4	2.84%
Q24. Is financial payment to your participation in community research?	32	22.54%	33	23.24%	34	23.94%	28	19.72%	15	10.56%

## Data Availability

Not applicable.

## Appendix A

RCHDR Community Engagement Core Community Trust Survey.
